# Severe immune-mediated myocarditis caused by sintilimab combined with gemcitabine: a case report and literature review

**DOI:** 10.3389/fcvm.2025.1559173

**Published:** 2025-04-09

**Authors:** Haixia Yang, Menglu Sun, Xiaosha Zhou, Yaxuan Han, Shanshan Zhang, Kelin Zhang, Xiaoyan Zhang

**Affiliations:** ^1^ Department of Radiation Oncology, Xi'an Chest Hospital Affiliated to Northwest University, Xi'an, China; ^2^ Department of Cardiovascular Medicine, Xi'an Chest Hospital Affiliated to Northwest University, Xi'an, China

**Keywords:** lung cancer, sintilimab, immune myocarditis, multidisciplinary collaboration, delayed diagnosis, case report

## Abstract

Following the approval of sintilimab for lung cancer immunotherapy in China in June 2021, real-world clinical practice has confirmed its efficacy and safety. Although there have been limited reports of immune-related myocarditis associated with sintilimab, no fatal cases have been reported to date. This case report focuses on a 71-year-old male patient with lung squamous cell carcinoma who developed severe immune-mediated myocarditis after receiving sintilimab in combination with gemcitabine. The patient presented with immune myocarditis combined with acute myocardial infarction. Due to delayed diagnosis, the outcome was unfavorable. This case is a warning to clinicians for early identification, rapid diagnosis and standardized use of glucocorticoids and immunosuppressants in sindilizumab induced myocarditis and emphasize the importance of multidisciplinary collaboration in managing such rare but serious adverse events.

## Introduction

In recent years, immunotherapy has emerged as a crucial treatment modality for lung cancer, providing new hope by blocking tumor immune escape and activating the body's immune system. Immune checkpoint inhibitors such as PD-1 and PD-L1 have significantly improved treatment outcomes (1). However, immune myocarditis, a rare but fatal adverse reaction, poses a significant challenge to immunotherapy. The incidence of ICI-related myocarditis ranges from 0.27 to 1.14% (2), and it is often severe and life-threatening. The reported incidence of ICIs-related myocarditis is low (<1%), but it can be life-threatening with a mortality rate of up to 60% (3, 4). Early diagnosis is difficult, especially in patients with concurrent cardiovascular diseases, leading to misdiagnosis and missed diagnoses (5). Sintilimab, a humanized monoclonal antibody against PD-1, was approved in China in 2021 for treating non-small cell lung cancer. It effectively blocks the interaction between PD-1 and PD-L1 and PD-L2, enhancing anti-tumor activity. While generally safe, reports of immune myocarditis are limited, and no fatal cases have been reported (6–11). We conducted an extensive search in PubMed, Web of science, CNKI and Wanfang databases by employing the keywords “sintilimab”, “lung cancer”, and “myocarditis”. Through this meticulous search process, a total of seven case reports related to sintilimab induced autoimmune myocarditis in lung cancer patients were carefully selected. It is notable that all the cases demonstrated either significant improvement or complete cure following proactive and aggressive treatment (see Table 1). This paper specifically presents a case where a patient with lung squamous cell carcinoma and underlying cardiovascular diseases experienced a severe immune-related myocarditis that tragically led to death after receiving the combination therapy of sintilimab and gemcitabine. The primary objective of presenting this case is to heighten clinicians' awareness and comprehension of this exceptionally rare complication. Moreover, it aims to offer valuable guidance for early diagnosis and the implementation of appropriate treatment strategies, with the ultimate goal of enhancing patient outcomes and prognosis.

**Table 1:** Characteristics of reported cases of sintilimab-induced myocarditis in lung cancer patients.

Author, year	Age/sex	PT/stage	ICI and Concomitant drugs	Onset of myocarditis	Clinical presentation	ADR	Diagnostic method	Cardiac biomarkers	ECG	Treatment
Lin Y et al., 2022	66/M	Ade/IV	TC + Sintilimab	3 weeks after 1 cycle	Chest pain, shortness of breath	Myocarditis	ECG, UCG, CAG	TnI, CK, CK- MB, NT-proBNP↑	V5 - V9 ST↑	MP, IG
Chen X et al., 2024	33/F	Ade/IV	PC + Sintilimab	2 days after 3 cycles	Shortness of breath, productive cough	Myocarditis	ECG, Chest CT	α - HBDH, cTn, CK-MB, NT-proBNP↑	Tachycardia	MP, De
Bi H et al., 2021	68/M	SCC/Ⅲb	TC + Sintilimab	6 days after 3 cycles	Productive cough, progressive dysphagia	Myocarditis	ECG, CAG	CK, CK-MB, hs-cTnl, BNP↑	CRBBB, Ⅱ°AVB, diffuse ST segment depression	MP
Xing Q et al., 2020	68/M	Ade/NA	Sintilimab	4 days after 2 cycles	Fatigue, myalgia, shortness of breath, progressive muscle weakness	Myocarditis, myositis, rhabdomyolysis	ECG, UCG	CPK, Myo, TnT, anti-AChR-Ab↑	CRBBB, Ⅲ°AVB	MP, IG, Mestinon, PE, TPM
Xia J et al., 2024	66/M	Ade/IVb	PN + Sintilimab	2 weeks after 2 cycles	Double eyelid ptosis, chest tightness	Myocarditis, hepatitis, pneumonia	ECG, UCG, CTA, Chest CT	CK-MB, Myo, ultra-TNI, NT-proBNP, AST, ALT↑	CRBBB, LAH, LAD, ST-T changes	MP, IG
Hu Y et al., 2023	60/M	SCC/ⅠA3	TC + Sintilimab	after 2 cycles	No symptoms	Myocarditis	CTA, ECG, UCG, CMR, Chest CT, myocardial biopsy	TnT, NT-proBNP↑	Normal sinus rhythm	MP
Zhang Y et al., 2023	58/M	Ade/IVb	PC + Sintilimab	after 1cycle	No symptoms	Myocarditis, myasthenia gravis, myositis	ECG, UCG, CAG, MPI, CMR, EMG	hsTNI, CK, CK-MB, Myo↑	Sinus rhythm, type A pre-excitation syndrome	MP
The present case report	71/M	SCC/T_4_N_3_M_x_	G + Sintilimab	17 days after 1cycle	Shortness of breath, cough, yellow sputum, fatigue, bilateral knee pain	Myocarditis	ECG, UCG, CAG	BNP, CK-MB, ultra-Tnl, Myo↑	Abnormal Q waves, ST-segment elevation	MP, IG

PT, pathological types; ICI, immune checkpoint inhibitors; M, male; Ade, adenocarcinoma; ECG, electrocardiograph; UCG, ultrasonic cardiogram; CAG, coronary arteriography; CMR, cardiac magnetic resonance; F, female; CTA, computed tomography angiography; SCC, squamous cell carcinoma; IG, immunoglobulin; MP, methylprednisolone; CRBBB, complete right bundle branch block; AVB, atrioventricular block; LAH, left anterior hemiblock; LAD, left axis deviation; PE, plasma exchange; ST, sinus tachycardia; APB, atrial premature beats; AT, atrial tachycardia.

## Case presentation

A 71-year-old male was diagnosed with left superior lobe squamous cell carcinoma (cT4N3Mx) on September 20, 2023. Genetic or PDL-1 testing was not carried out. From October to November 2023, he received radiotherapy for lung and mediastinal lesions, with a target dose of 54Gy and an average cardiac dose of 3.4Gy, achieving a partial response (PR). From December 2023 to July 2024, he underwent 5 cycles of albumin-bound paclitaxel + cisplatin chemotherapy, among which the 4th cycle was combined with recombinant human endostatin (Endu). Due to cardiac reactions related to Endu, the treatment was not completed, and the efficacy was stable disease (SD). In October 2024, the disease progressed (PD), resulting in gemcitabine + sintilimab treatment starting on October 21, 2024. Baseline assessment before immunotherapy revealed normal myocardial injury markers and BNP. ECG showed sinus rhythm, unbiased electrical axis, low extremity conduction voltage, complete right bundle branch block, and prolonged Q-TcF interval. Cardiac ultrasound indicated reduced left ventricular diastolic relaxation function but normal systolic function (EF 63%).

**Figure 1:**
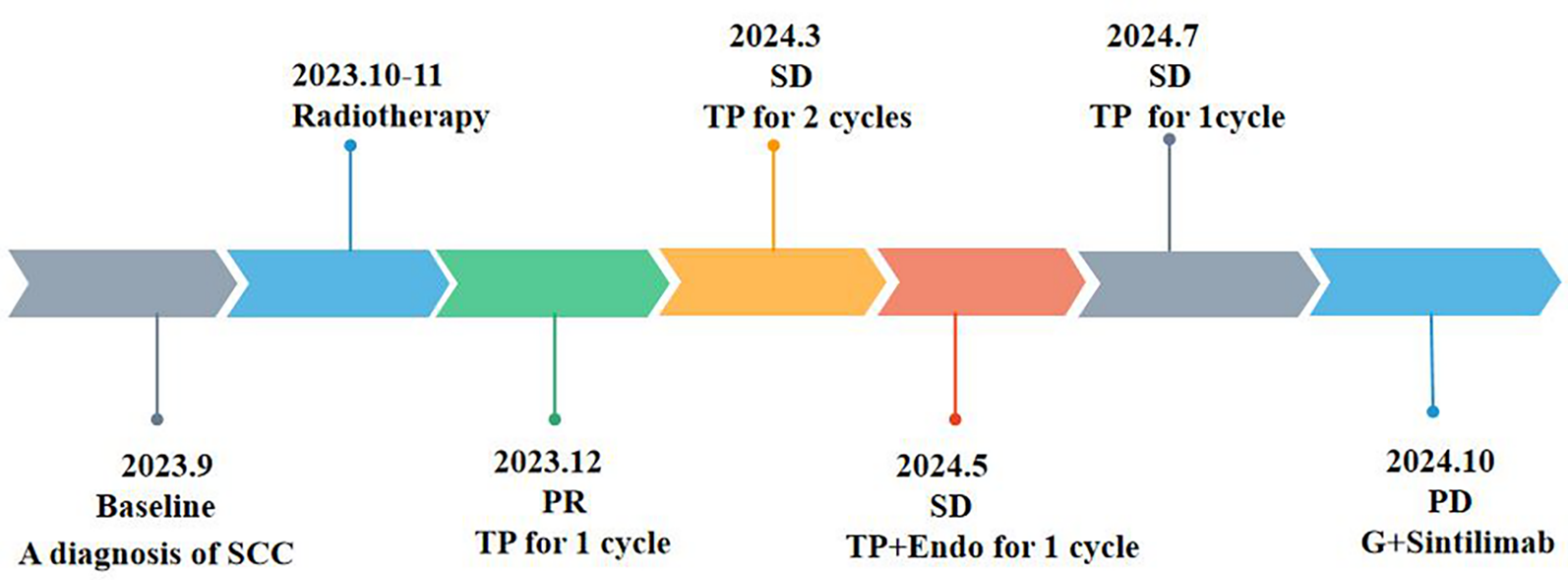
Chronology of integrated antitum or therapy.

**Figure 2:**
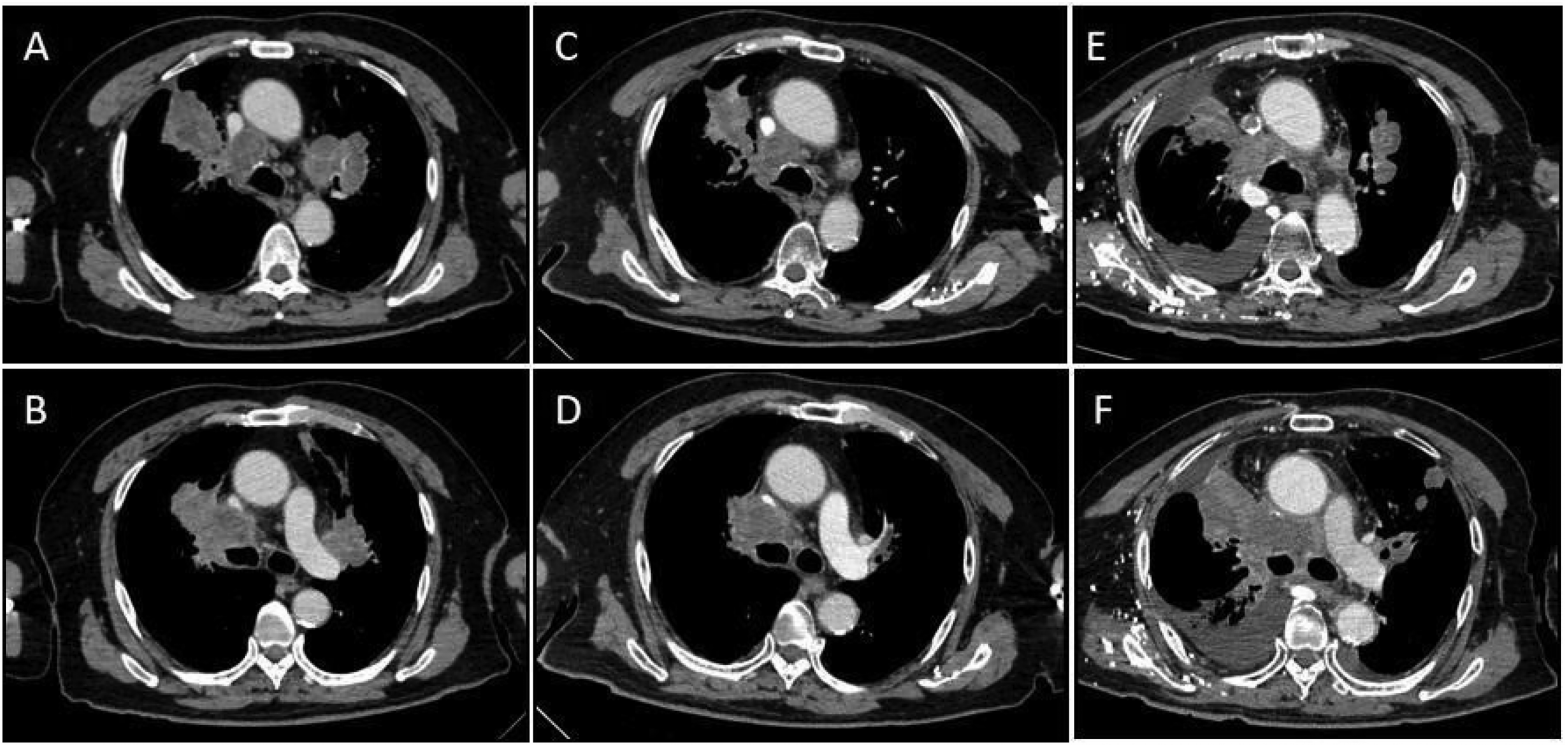
Comparison of chest CT images at different stages. **(A,B)** Initial diagnosis, **(C,D)** post-radiotherapy, **(E,F)** pre-immunotherapy with sintilimab.

Thyroid function was not tested. The anti-tumor situation is presented in Figure 1, and chest CT images are shown in Figure 2. Previous medical history. The patient has a documented history of cerebral infarction for over a decade, characterized by mild right-sided limb weakness and intermittent numbness in the upper extremities. In May 2024, the patient received a clinical diagnosis of coronary heart disease without undergoing coronary computed tomography angiography (CTA).

The patient is on long-term management with aspirin and atorvastatin.

**Figure 3:**
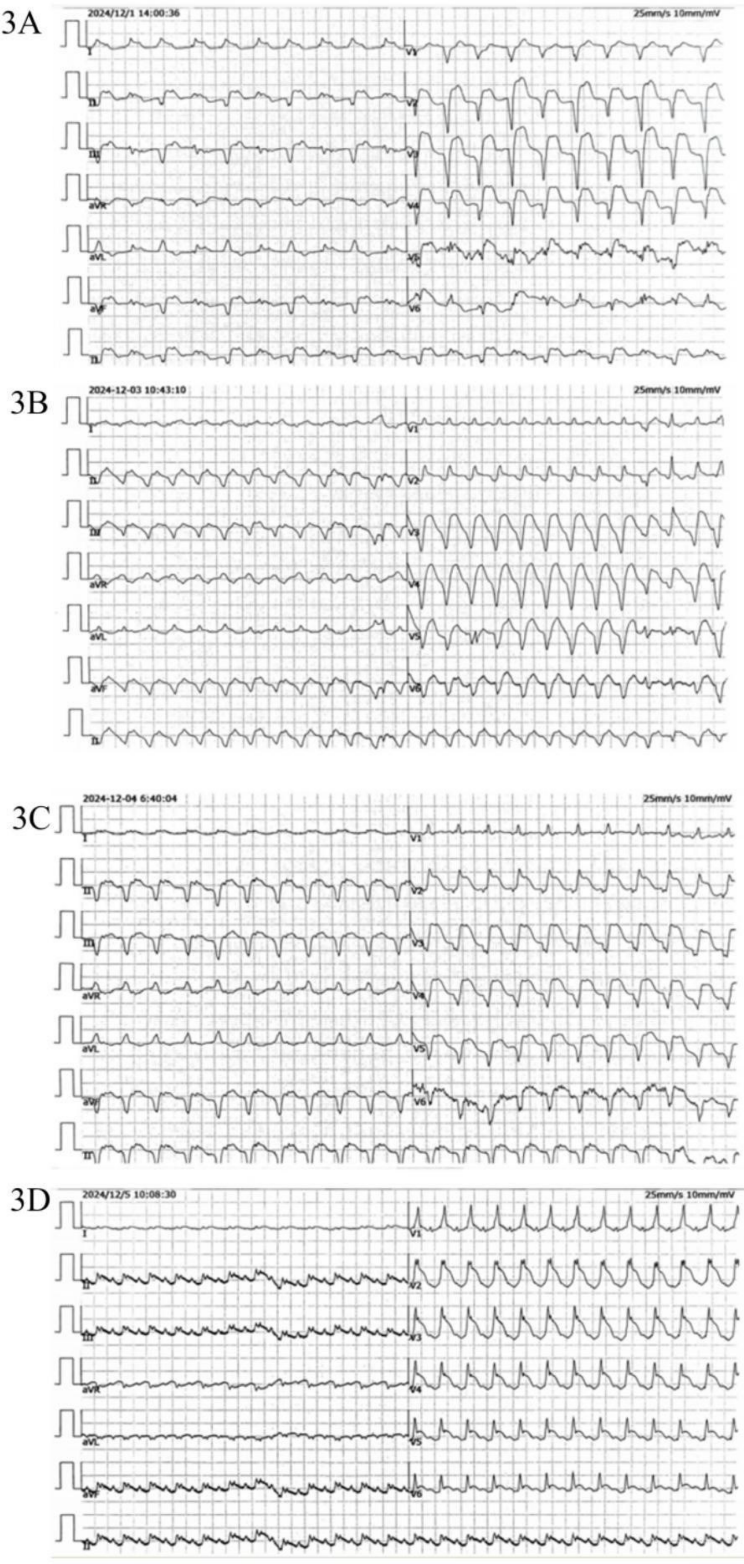
ECG **(A)** extensive ST-T changes are observed in leads II, III, aVF, and V1-V6, with the presence of abnormal Q waves and ST segment elevation (2024/12/1). **(B,C)** Ectopic rhythm mean ventricular rate, wide QRS tachycardia. **(D)** Sinus rhythm with sinus tachycardia, complete right bundle branch block, and widespread STT changes across multiple leads.

**Figure 4:**
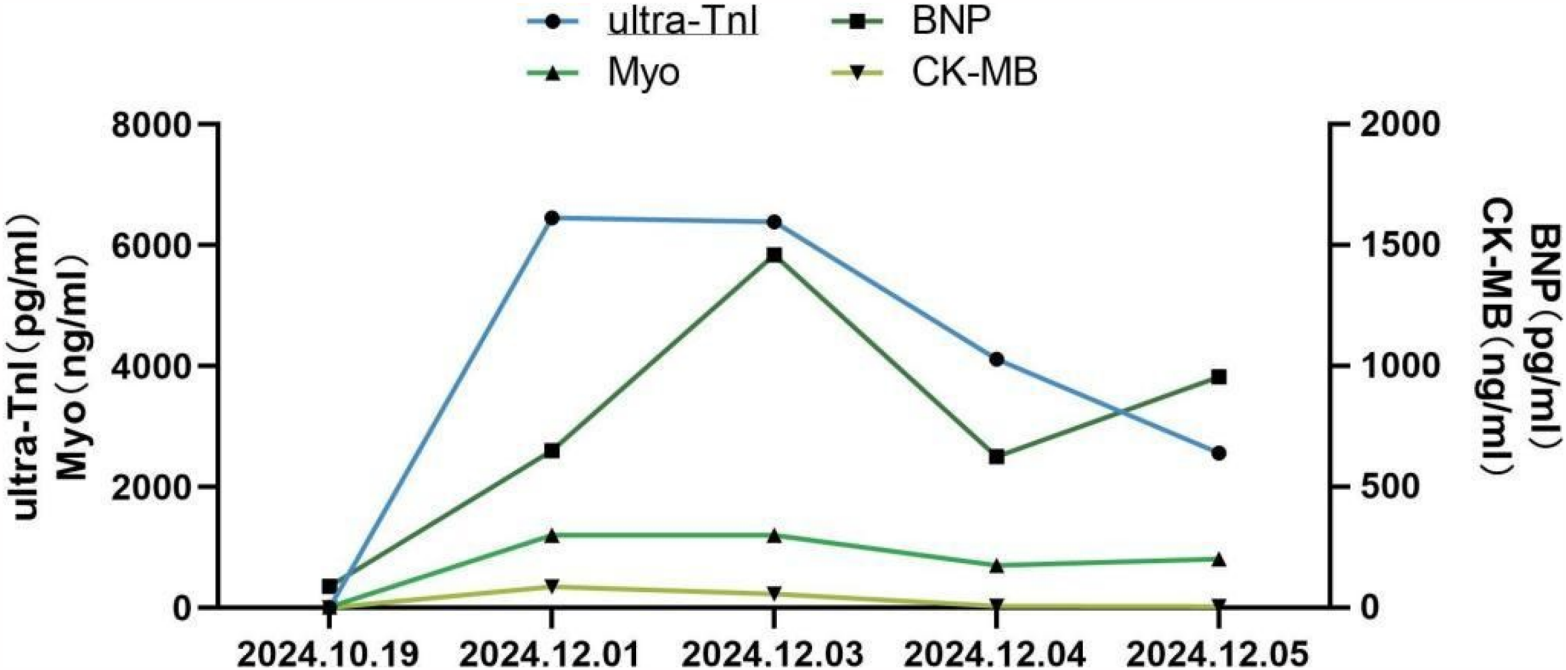
Alterations in serum myocardial markers during myocarditis ultra-TnI, high sensitivity cardiac troponin I; BNP, B-type natriuretic peptide; Myo, myoglobin; CK-MB, creatine kinase MB.

**Figure 5:**
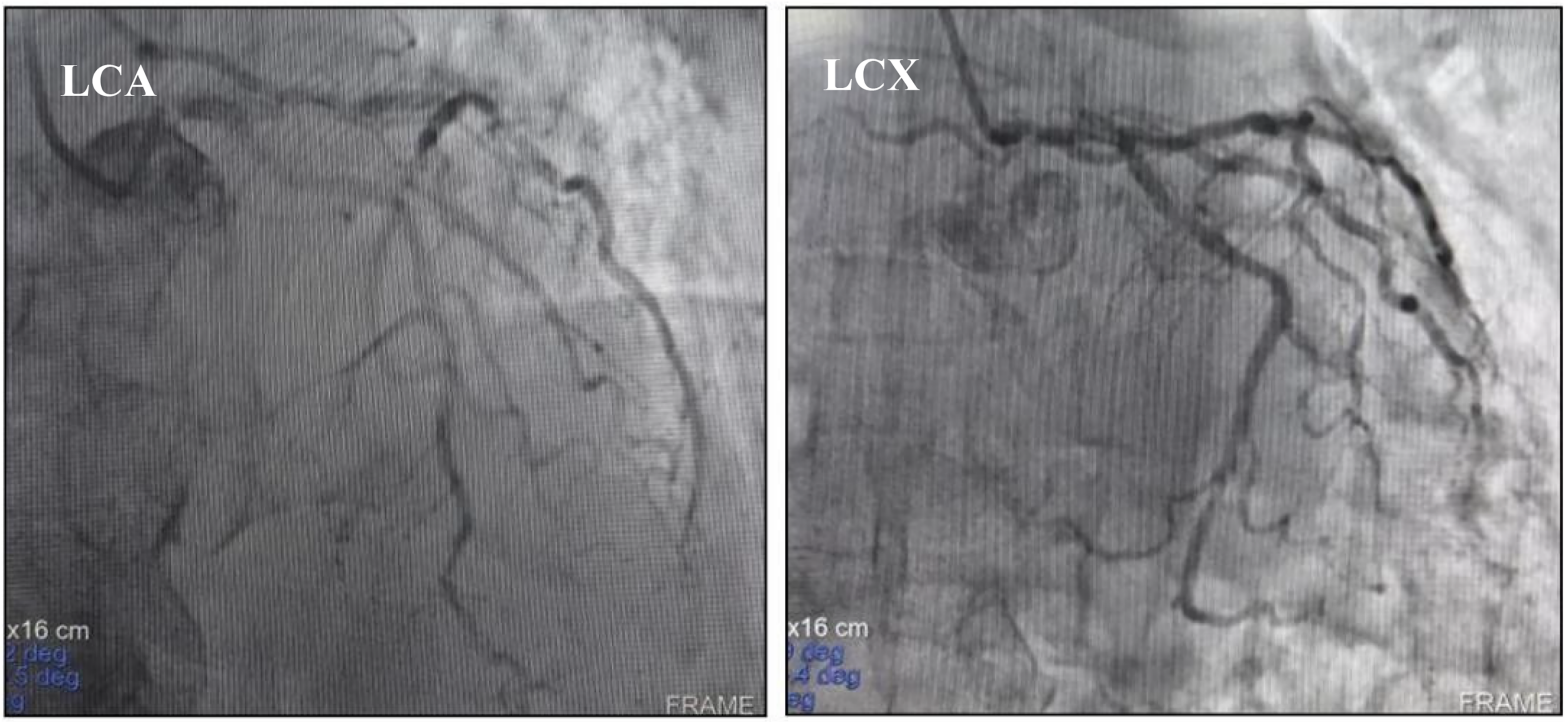
Coronary angiography did not support coronary artery disease as the cause of acute myocardial infarction. LCA, left coronary artery; LCX, proximal circular artery.

On November 28, 2024, the patient manifested shortness of breath, accompanied by cough, yellow sputum, fatigue, and bilateral knee pain. However, these symptoms were initially not given sufficient attention. On December 1, 2024, the patient's shortness of breath deteriorated significantly, resulting in emergency hospital admission. The electrocardiogram indicated abnormal Q waves in leads II, III, aVF, and V1-V6, along with ST-segment elevation, suggesting acute myocardial infarction (see Figure 3A). There was a reduced stroke amplitude in the anterior wall, posterior wall, and anterior interventricular septum. Markers of myocardial injury were significantly elevated, such as hypersensitive troponin at 6456.6 pg/ml (reference range: 0–34.2 pg/ml), BNP at 650.8 pg/ml (reference range: 0–100 pg/ml), and myoglobin > 1,200 ng/ml (reference range: 0–146.9 ng/ml) (see Figure 4). Considering the potential for coronary artery stenosis to cause acute myocardial infarction (AMI), a consultation with a cardiologist was sought. However, immune-related myocarditis could not be ruled out. Given that corticosteroid administration in AMI may compromise infarct healing and repeated high-dose corticosteroid therapy could increase the risk of ventricular rupture or aneurysm formation, initial treatment comprised antiplatelet agents (aspirin, clopidogrel), cardioprotective medications (Coenzyme Q10, polarizing solution, nicotinamide), ventricular rate control drugs (metoprolol tartrate, esmolol), heart failure management drugs (recombinant human brain natriuretic peptide), and anti-infective agents (piperacillin-tazobactam, meropenem), without the use of corticosteroids. Additionally, anticoagulant therapy was not initiated owing to the patient's elevated risk of gastrointestinal bleeding secondary to long-term aspirin use. On December 2, 2024, the patient's symptoms of chest tightness and shortness of breath persisted. Coronary angiography was carried out, revealing a 40% stenosis in the left main trunk, a 50% stenosis in the proximal intima of the left anterior descending branch with distal antegrade blood flow at TIMI-3 grade, a 50% stenosis in the proximal circumflex branch, an 85% stenosis in the distal circumflex branch with distal antegrade blood flow at TIMI-3 grade, and patency in the right coronary artery. Although coronary angiography showed no significant stenosis requiring a stent placement (see Figure 5), the patient's ECG and cardiac indicators suggested acute myocardial infarction. Further evaluation of the cause of acute myocardial infarction using IVUS, OCT, or FFR was warranted. This type of myocardial infarction is associated with microvascular dysfunction, coronary artery spasm, plaque rupture, or other rare conditions. The patient may have had acute myocardial infarction combined with immune myocarditis.

As a result, treatment was promptly initiated with 20 g of intravenous human immunoglobulin and 360 mg of methylprednisolone sodium succinate. On December 3, 2024, the patient presented with a poor mental state and intermittent chest tightness as well as shortness of breath. The BNP levels rose to 1,460.7 pg/ml, a marked increase compared to December 1, 2024. The hypersensitive troponin remained at 6,387.5 pg/ml, while myoglobin exceeded 1,200 ng/ml (see Figure 4). Echocardiography revealed an EF of 51% using the biplane method, while Pulse Doppler demonstrated an E/A ratio less than 1. These findings suggest a reduction in left ventricular diastolic function. The dosage of methylprednisolone sodium succinate was adjusted to 240 mg twice daily, and the immunoglobulin shock therapy was continued. At 10:12 AM on December 3, 2024, the patient experienced a sudden loss of consciousness accompanied by hypotensive shock. Immediate interventions included initiating mechanical ventilation support, performing endotracheal intubation, central venous catheterization, urinary catheter insertion, invasive ventilator-assisted respiration, and administration of vasopressors. By 11:36 AM, the patient's vital signs had stabilized; however, he remained in an unconscious state. High-dose corticosteroids, intravenous immunoglobulin, antimicrobial agents, and anti-shock measures were continued. As of December 4, 2024, the patient remained intubated and dependent on mechanical ventilation without regaining consciousness. Serial measurements revealed a decrease in BNP to 625.6 pg/ml, hypersensitive troponin to 4,117.3 pg/ml, and myoglobin to 702.3 ng/ml, all of which were lower than previous levels (see Figure 4). An electrocardiogram (ECG) revealed an ectopic rhythm, left axis deviation, wide QRS tachycardia (excluding ventricular tachycardia), and a prolonged QTcF interval (see Figure 3C). On December 5, 2024, the hypersensitive troponin level further decreased to 2,561.4 pg/ml; however, both BNP (956.4 pg/ml) and myoglobin (811.5 ng/ml) remained elevated compared to previous levels (see Figure 4). The ECG findings included sinus rhythm, normal electrical axis, sinus tachycardia, complete right bundle branch block, limb lead low voltage, and widespread ST-T changes. Please refer to the clinical context provided in Figure 3D. Due to the family's decision to discontinue treatment, the patient was discharged from the hospital on December 5, 2024, and subsequently passed away on the same day.

## Discussion

This paper reports on the diagnosis, treatment, and outcome of grade 4 immune-related myocarditis in a patient with squamous cell carcinoma of the lung after undergoing chemoradiotherapy combined with sintilimab and gemcitabine. It typically demonstrates the severity, complexity of diagnosis, and challenging nature of the treatment of this disease. We reflect upon the deficiencies in the diagnosis and treatment process, propose optimization strategies for risk assessment, diagnosis and treatment, and caution clinicians to be highly vigilant about this rare complication throughout the entire process of lung cancer immunotherapy, emphasizing early identification and precise intervention to enhance patient prognosis. In this case, the patient developed immune-associated myocarditis 38 days following administration of sintilimab. The underlying mechanism is likely related to the activation of T lymphocytes by sintilimab. Specifically, T lymphocytes activated by immune checkpoint inhibitors (ICIs) may recognize shared myocardial antigens, resulting in lymphocytic infiltration of the myocardium and subsequent myocarditis (8). When employing ICIs to treat lung cancer patients, clinicians should conduct a comprehensive evaluation and remain highly vigilant regarding the occurrence of immune-associated myocarditis (12, 13). This patient had a 50-year history of smoking and documented histories of cardiovascular and cerebrovascular diseases, coronary heart disease, as well as prior chest radiotherapy and chemotherapy before undergoing immunotherapy. Collectively, these factors constitute significant risk factors for immune-mediated myocarditis. Hence, the risk assessment of immune myocarditis before treatment with sintilimab was inadequate. Furthermore, neither the patients nor their family members demonstrated a robust understanding of monitoring symptoms potentially indicative of myocarditis, such as chest tightness, shortness of breath, dyspnea, and fatigue, following immunotherapy. Consequently, they were unable to seek medical attention promptly, leading to missed opportunities for early intervention and delayed treatment (14). Injury markers, complicating the diagnostic process. In this case, autoimmune myocarditis coexisted with acute myocardial infarction, which increased the difficulty of diagnosis and treatment. This observation is in line with previous reports, which suggest that immune-associated myocarditis often lacks specific clinical, laboratory, or radiographic diagnostic criteria and must be carefully differentiated from other cardiovascular diseases (15, 16, 25). During the diagnosis, a comprehensive assessment of the patient's medication history, clinical symptoms, cardiac biomarkers, ECG findings, and imaging results is indispensable. Myocardial biopsy should be contemplated if necessary to confirm the diagnosis (17, 24). In this case, the patient rapidly deteriorated after admission, experiencing hemodynamic instability and shock. Endotracheal intubation and resuscitative measures were performed, but dependence on mechanical ventilation made an emergent cardiac biopsy impossible.

The suboptimal treatment outcomes of this patient can be ascribed to two main factors. Firstly, the inherent severity of immune-related myocarditis is marked by its rapid progression. Once it occurs, a considerable number of cardiomyocytes are damaged, causing a sharp decline in cardiac function. Secondly, early diagnosis presents considerable challenges, as myocardial inflammation often reaches an advanced stage by the time it is detected. As a result, the optimal window for intervention is missed, and the current therapeutic options remain limited. While immunomodulatory agents can partially alleviate immune hyperplasia, they are insufficient in completely reversing multifactorial myocardial damage, leading to an adverse prognosis. This highlights the perilous nature and therapeutic complexity of immune-related myocarditis, which is in line with the previously reported high mortality rates (18, 19).

Once immune-related myocarditis is diagnosed, high-dose corticosteroids are considered as the first stage of immunosuppressive therapy (20). Intravenous Methylprednisolone of 1,000 mg/day is recommended as a pulse dose, usually for three days, followed by 1 mg/kg daily either intravenously or orally. The American Society of Clinical Oncology guidelines recommend a tapering of at least 4–6 weeks.

However, specific tapering should be tailored on a case-by-case basis (21). Zhang et al. found that the time of initiation of steroid treatment impacted MACE-free survival, whereby patients receiving corticosteroids within 24 h, regardless of dosage, showed the best outcome, and patients receiving corticosteroids after 72 h, regardless of dosage, showed the worst outcome (22). In this case, the delayed diagnosis of immune-related myocarditis led to missed opportunities for optimal treatment. High-dose methylprednisolone sodium succinate and intravenous immunoglobulin were not administered until five days after symptom onset. Despite aggressive therapeutic interventions, the patient's condition rapidly deteriorated, resulting in a fatal outcome. Furthermore, earlier consideration of immunosuppressants such as infliximab, mycophenolate mofetil (MMF), or tacrolimus, either individually or in combination, along with timely life support treatment, might have potentially improved clinical outcomes. Additionally, insufficient initial dosage of glucocorticoids indicate that clinicians need to enhance their understanding of ICIS-related deadly myocarditis. In summary, preimmunotherapy risk assessment is crucial for elderly patients with a history of cardiovascular and cerebrovascular diseases as well as thoracic radiotherapy and chemotherapy. Timely diagnosis of immune-associated myocarditis is essential to prevent missed treatment opportunities. Furthermore, in the event of deteriorating conditions, it is imperative to promptly optimize the treatment plan and implement multiple measures concurrently. Future research should be focused on further exploring the pathogenesis of immune-related myocarditis and seeking more accurate and effective early diagnostic approaches and personalized treatment plans. In the face of complex diseases, it is crucial to strengthen multidisciplinary collaboration among oncology, cardiology, rheumatology, and other departments to lower the mortality rate of severe immune myocarditis (5, 23).

## Data Availability

The datasets presented in this article are not readily available because of ethical and privacy restrictions. Requests to access the datasets should be directed to the corresponding author/s.
